# An unusual cause of chronic low back pain: ochronosis

**DOI:** 10.11604/pamj.2017.26.81.11754

**Published:** 2017-02-21

**Authors:** Zeineb Alaya, Anis Mzabi

**Affiliations:** 1 Department of Rheumatology, Farhat Hached Hospital, Faculty of Medicine of Sousse, Sousse, Tunisia; 2 Department of Internal Medicine, Sahloul Hospital, Faculty of Medicine of Sousse, Sousse, Tunisia

**Keywords:** Ochronosis, calcification disc, low back pain

## Image in medicine

A 45-year-old man presented with low back pain evolving since 8 years associated sometimes with a radicular radiation. The clinical examination found a patient in good general condition, a loss of the lumbar lordosis, an exaggeration of the dorsal kyphosis with stiffness of the lumbar spine. The cutaneous-mucosal examination showed a brownish appearance of the ears, conjunctiva, and lower eyelids. In biology there was neither inflammatory syndrome nor perturbation of the phosphocalcic balance. The standard radiographs of the lumbar spine (A) and dorsal spine (B) revealed multiple disc calcifications associated with large staggered disc clumps and discal empty images. Due to mucocutaneous signs, normality of biology and the radiological aspect, the diagnosis of ochronosis was suspected and confirmed by the test of blackening of the urine to the ambient air, the results of the cutaneous biopsy showed pigmented deposits yellow ocher and brownish in the dermis and the dosage of alkaptonuria. Symptomatic treatment resulted in moderate improvement. The family survey did not reveal any similar cases. Ochronosis or alkaptonuria is a rare autosomal recessive disorder of tyrosine metabolism. Deficiency of homogentisate 1,2 dioxygenase results in accumulation of oxidized homogentisic acid in the connective tissues of the skin, eyes and ears, musculoskeletal system, and cardiac valves, and in urolithiasis. Ochronosis is of late onset, responsible for the rachis of extended disc calcifications with maximum spinal fusion. It is the cause of degenerative arthropathy. Early diagnosis and screening of this disease are then of great interest, especially for genetic counseling.

**Figure 1:**
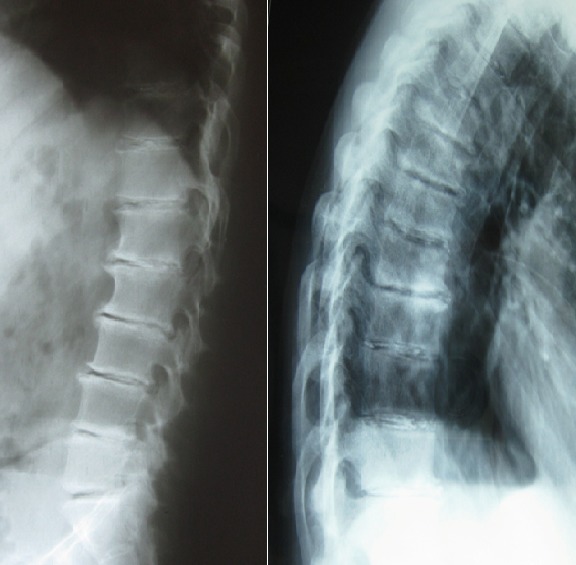
The standard radiographs of the lumbar spine (A) and dorsal spine (B) revealed multiple disc calcifications associated with large staggered disc clumps and discal empty images

